# Pricing of Shanghai stock exchange 50 ETF options based on different volatility models

**DOI:** 10.1371/journal.pone.0288266

**Published:** 2023-12-07

**Authors:** Qingchun Wu, Xiaoping Kuang, Binhong Wu, Xuhong Xu

**Affiliations:** 1 Tan Siu Lin Business School, Quanzhou Normal University, Quanzhou, China; 2 College of Computer and Information Sciences, Fujian Agriculture and Forestry University, Fuzhou, China; Universiti Malaysia Sabah, MALAYSIA

## Abstract

On March 15, 2022, the volume of trade of the Shanghai Stock Exchange (SSE) 50 ETF option contracts and the CSI 300 ETF option contracts exceeded 10 million for the first time, of which 5,707,400 50 ETF options were traded, and SSE 50 ETF options, as the main force, has become one of the most active ETF option varieties in the world after seven years of vigorous development. The SSE 50 ETF options receive highlights in risk-free arbitrage, hedging, risk management and other aspects. In order to give full play to the function of the SSE 50 ETF options, it is necessary to conduct studies on their pricing. This paper adopts the traditional classical models for pricing European-style options, the BSM model and the volatility model, to price call options and put options of the SSE ETF, and meanwhile analyzes the volatility of the SSE 50 ETF. The empirical results suggest that (1) the volatility of SSE 50 ETF has a weak leverage effect or no leverage effect, which converges with the existence of the inverse leverage effect of the SSE index; (2) the BSM model will underestimate the price of SSE 50 ETF options and is only ideal for pricing in-the-money (ITM) options; while out-of-the-money (OTM) options are highly influenced by time value and therefore cannot be accurately priced.

## Introduction

The development of the financial derivatives market has become a significant influencer of the global economy. As important financial instruments, options are derivatives on the basis of futures with features such as short selling mechanism, hedging, and leverage, which help to hedge the market risks, enhance resource allocation efficiency,and accelerate the transformation of the real economy. Therefore options are indispensable as a risk management tool for the mature capital market. The core issue of options is pricing. Reasonable price discovery is the basis of all transactions, such as arbitrage and hedging strategies. Due to the late progress of China’s capital market, the relatively immature market construction and the speculative nature of investors, options products have been widely used. However, in the context of increasingly frequent options trading, options provide investors, risk managers, and speculators with trading opportunities and management tools. Frequent speculative operations can make options risks complex and changeable. The reasonable pricing of an underlying option will help reduce market speculation so that investors can invest more rationally and China’s financial market can develop steadily and persistently.

This paper’s core issue is finding a good and efficient option pricing scheme for the Chinese market to help investors make more reasonable investment decisions based on information rather than noise. On the one hand, western countries have a more mature and systematic theoretical basis and pricing mechanism. However, the applicability of this system to the current market in China requires further exploration. On the other hand, the 50ETF option contract, the first exchange-traded option product in China, has gained massive progress in the transaction after seven years of rapid development. In 2021, the average daily trading volume of the option contract will be 2.6 million. From the proportion of call options and put options, the trading volume of call options is enormous, accounting for 55% of the total volume. Currently, the 50ETF option contract has become one of the major ETF options in the world. As a result, the studies of option pricing attach critical meanings to the global options market.

In order to comprehensively and accurately study China’s option pricing scheme and reasonably use options to avoid and reduce risks, this paper selects the closing prices of the SSE 50ETF option contract from February 9, 2015, to April 27, 2022, using ARMA - GARCH model and ARMA - APARCH model to model and predict future volatility. At the same time, in order to test the effect of the model on predicting volatility, the real market prices of options are compared to the theoretical price of the BSM option pricing model. Finally, the volatility model is evaluated based on the comparison.

The empirical results show that: (1) The coefficient estimation of the APARCH model is not significant, and there is a weak leverage effect in the logarithmic yield of the SSE 50ETF, which is basically consistent with the market characteristics of China’s SSE Index; (2) The BSM option pricing model combined with GARCH model has a reasonable pricing effect on real options, which is consistent with the options market price. The assumptions of the BSM model do not affect its application in practice; (3) The pricing effect of options is only related to the value state and is not affected by economic turbulence. This pricing scheme can stand the test of the market.

### Literature review

In terms of option pricing, western academia leads the research area. Bachelier first defined option pricing in 1900, setting the stage for subsequent research on option pricing. Since then, various pricing models have been proposed, but most are unacceptable because assumptions fail to conform to reality.

Until the 1970s, with the vigorous development of the option market, option pricing achieved a breakthrough. In 1973, Fisher Black and Myron Scholesbuilt a portfolio with a mathematical method where the portfolio’s characteristics of profit and loss completely reflected those of the option at expiration regardless of the changes of the underlying asset price in the future [1]. The pricing formula of European-style stock calls was derived based on the portfolio. This method worked well in practice. However, this pricing formula is subject to strict assumptions, and as a result, the pricing fails to hold water. Many scholars, therefore, proposed revised models based on this pricing formula. Merton’s revision is the most convincing because it not only considers dividend payment, which aligns with the actual situation but also overturns the assumption of a constant risk-free interest rate [2]. This revision finally forms the Black-Scholes-Merton model (BSM model). In 1976, Cox&Rossestablished the risk-neutral pricing theory, which assumes that all investors are risk neutral, and its core is to construct the risk-neutral probability [3]. Therefore, the risk-neutral pricing principle is closely related to the arbitrage-free equilibrium pricing principle. In order to make the BSM model applicable to American option pricing, Cox, Ross and Rubinsetein put forward the binomial option pricing model in 1979 [4].

This model inherited the general assumptions of the BSM model, which was characterized by looser assumptions, conformance to reality and calculation-friendliness. In 1979, Harrison and Kreps proposed martingale pricing based on the theory that the discount price process under the risk-neutral measure is a martingale or that the option price is equal to the expectation under the equal martingale measure after discounted by the risk-free interest rate [5].

In this paper, we selected the SSE 50ETF option to study option pricing. Since it is a European-style option, the BSM model is suitable because it has an exact pricing formula. However, the assumption that volatility is a constant in the model is not in line with reality. Therefore, applying this model to option pricing needs to predict volatility.

The application of the BSM option pricing model must predict volatility based on asset prices. After massive studies on volatility, scholars have proposed various volatility models. Engle put forward the ARCH model when addressing the British inflation rate and successfully built a systematic framework for volatility modeling [6]. It solved the problem of volatility estimation with an accurate reflection of the characteristics of asset volatility, laying the foundation for volatility prediction. Although ARCH is simple, it usually requires many parameters when portraying the volatility of asset prices, and estimating so many parameters is complex and inefficient, potentially causing overfitting chaos. In order to simplify the model, Bollerslev proposed the generalized ARCH model (GARCH) [7], which is more convenient and widely used than ARCH in empirical studies because it requires fewer parameters to be estimated and has better prediction accuracy than the ARCH model. The GARCH model can portray volatility aggregation, but it also has weaknesses. It cannot fully capture the leverage effect of volatility adequately. Therefore, Ding et al. proposed the class of APARCH models, i.e., asymmetric exponential autoregressive conditional heteroskedasticity [8]. However, other values of the parameter δ are not well explained except for some special values. The APARCH model is more flexible in the GARCH family of models and has become an important choice for volatility modeling [9].

Chinese scholars also researched option pricing with a focus on the application instead of theoretical innovation. Zhang Yuankun and Yang Hua used the historical volatility calculation formula and the BSM model to price the CSI 300 stock index options [10]. The results showed that the BSM model could accurately price the 300 stock index options. Lu Zhihong, while explaining the characteristics of the volatility of the SSE 50ETF, applied the GARCH(1,1) model to forecast the volatility, and the results showed that the GARCH(1,1) model had a good effect on the volatility prediction of the SSE 50ETF [11]. Guan Lushan applied the simple GARCH(1,1) model and the stochastic volatility (SV) model combined with the BSM model to make a comparative analysis of prediction and found that the prediction effect of the GARCH model was better than that of SV model [12].

Therefore, this paper selects the BSM model to price the option contract, uses the simple GARCH(1,1) model and the APARCH(1,1) model to deal with the leverage effect to predict the volatility required by the option pricing model, and analyzes the volatility of the SSE 50 ETF.

### Model introduction

The ideas for option contract pricing in this paper are as follows: (1) establishing the ARMA mean equation of return rate; (2) adopting GARCH (1,1) model and APARCH (1,1) model dealing with leverage effect to forecast the volatility required by the option pricing model; (3) using predicted volatility to replace the constant volatility in the BSM pricing formula for pricing effect analysis. The details are as follows:

#### Arma model

The first step in establishing a volatility model is to set up the mean rate of return equation. When determining the order of the simple AR or MA model, the high-order model may be selected, which undoubtedly complicates the parameter estimation. Therefore, the ARMA model proposed by Box et al. [13] is used. It can be seen that the ARMA model is a combination of AR and MA models, and the number of parameters to be estimated by the model should be controlled within a small range. A brief introduction to the ARMA model is therefore necessary. The general ARMA(p, q) model can be expressed as:

$$x_{t}=\phi_{0}+\sum_{i=1}^{p}\phi_{i}x_{t-i}+a_{t}-\sum_{i=1}^{q}\theta_{i}a_{t-i}$$ (1)

The identification of the ARMA model can rely on AIC or BIC information criteria, selecting AIC(BIC) minimum model.

#### Volatility model

GARCH (1,1) model and APARCH (1,1) model dealing with leverage effect are selected to forecast the volatility required by the option pricing model. The generalized autoregressive conditional heteroscedasticity (GARCH) model is Engle’s improvement of the ARCH model. The difference between the GARCH model and the ordinary regression model is that the GARCH model models the variance of error again. It has an excellent effect on the analysis and prediction of volatility. Therefore, such analysis can play a key guiding role in investors’ investment strategies, and its significance is higher than numerical analysis and prediction.

In a logarithmic yield sequence, we set *a*_*t*_ = *r*_*t*_−*μ*_*t*_ as moment new interest. The standard GARCH(m, s) model can be expressed as:

$$\left\{ \begin{array}{c} r_{t}=c_{1}+\sum_{i=1}^{R}\phi_{i}r_{t-i}+\sum_{j=1}^{M}\phi_{j}\varepsilon_{t-j}+\varepsilon_{t}\\ a_{t}=\sigma_{t}\varepsilon_{t}\\ \sigma_{t}^{2}=\omega +\sum_{i=1}^{m}\alpha_{i}a_{t-i}^{2}+\sum_{j=1}^{s}\beta_{j}\sigma_{t-j}^{2} \end{array}\right.$$ (2)

Where *ε*_*t*_ is white noise, ω > 0, α_*i*_ ⩾ 0, β_*j*_ ⩾ 0, 0 < $\sum_{i=1}^{max(m,s)}\left(\alpha_{i}+\beta_{i}\right)<1$。

Ding, Granger and Engle found that *a*_*t*_ has a strong autocorrelation [8]. That is the return on financial assets has a long memory.This phenomenon can be explained by conditional heteroscedasticity. Therefore, they proposed an asymmetric power autoregressive conditional heteroscedasticity (APARCH) model. The model assumes that the exponent of conditional variance is an undetermined parameter. Generally speaking, the form of the APARCH(m,s) model is:

$$\left\{ \begin{array}{c} r_{t}=\mu_{t}+a_{t},a_{t}=\sigma_{t}\varepsilon_{t},\varepsilon_{t}∼D(0,1)\\ \sigma_{t}^{\delta }=\omega +\sum_{i=1}^{m}\alpha_{i}{\left(|a_{t-i}|+\gamma_{i}a_{t-i}\right)}^{\delta }+\sum_{j=1}^{s}\beta_{j}\sigma_{t-j}^{\delta } \end{array}\right.$$ (3)

Among them, (0, 1) is a zero mean variance of a distribution unit, δ is a positive number, and other coefficients are adjusted to make volatility positive. In practice, we generally use the APARCH(1,1) model.

In the APARCH and the GARCH (1, 1) models, when δ = 2, the model is simplified to the TGARCH (1, 1) model. When δ = 2, γ = 0, the model is simplified GARCH (1, 1) model. When δ = 0, the model is simplified EGARCH (1, 1) model [9].

#### BSM model

The BSM model modified by Merton is widely known as the "B-S model." Based on ITO’s lemma, this model adopts the non-arbitrage pricing principle and deduces the option pricing formula. The BSM model has become the most widely used and popular option pricing model in the world due to the ingenious logic, precision and simple operation of the mathematical formula derived behind it. The option’s theoretical price can be obtained only by substituting relevant parameters. The theoretical price can be quickly calculated by computer software to provide a reference for investors. The assumptions of the BSM model are as follows:

1. The income from the underlying asset follows a lognormal distribution;
2. The risk-free rate is known and fixed during the option trading day;
3. The volatility of the underlying asset is constant;
4. Transaction costs are not considered;
5. Dividends are not considered (this assumption was later discarded);
6. The option cannot be exercised before the expiration date (i.e., the option is a European option).

Based on the above assumptions, the BSM option pricing formula is expressed as:

$$\left\{ \begin{array}{c} c=Se^{-q(T-t)}N(d_{1})-Ke^{-r(T-t)}N(d_{2})\\ p=Ke^{-r(T-t)}N({-d}_{2})-Se^{-r(T-t)}N(-d_{1}) \end{array}\right.$$ (4)

including:

$$\left\{ \begin{array}{c} d_{1}=\frac{\ln\left(\frac{S}{K}\right)+(r-q+\frac{\sigma^{2}}{2})(T-t)}{\sigma \sqrt{T-t}}\\ d_{2}=\frac{\ln\left(\frac{S}{K}\right)+(r-q-\frac{\sigma^{2}}{2})(T-t)}{\sigma \sqrt{T-t}} \end{array}\right.$$ (5)

where:

S—spot price of the underlying asset;

K—the strike price;

T—t—time to maturity;

σ—volatility of returns of the underlying asset;

r—risk-free rate;

q—dividend yield;

N()—cumulative distribution function of the standard normal distribution;

### Empirical results

**Fig 1:**
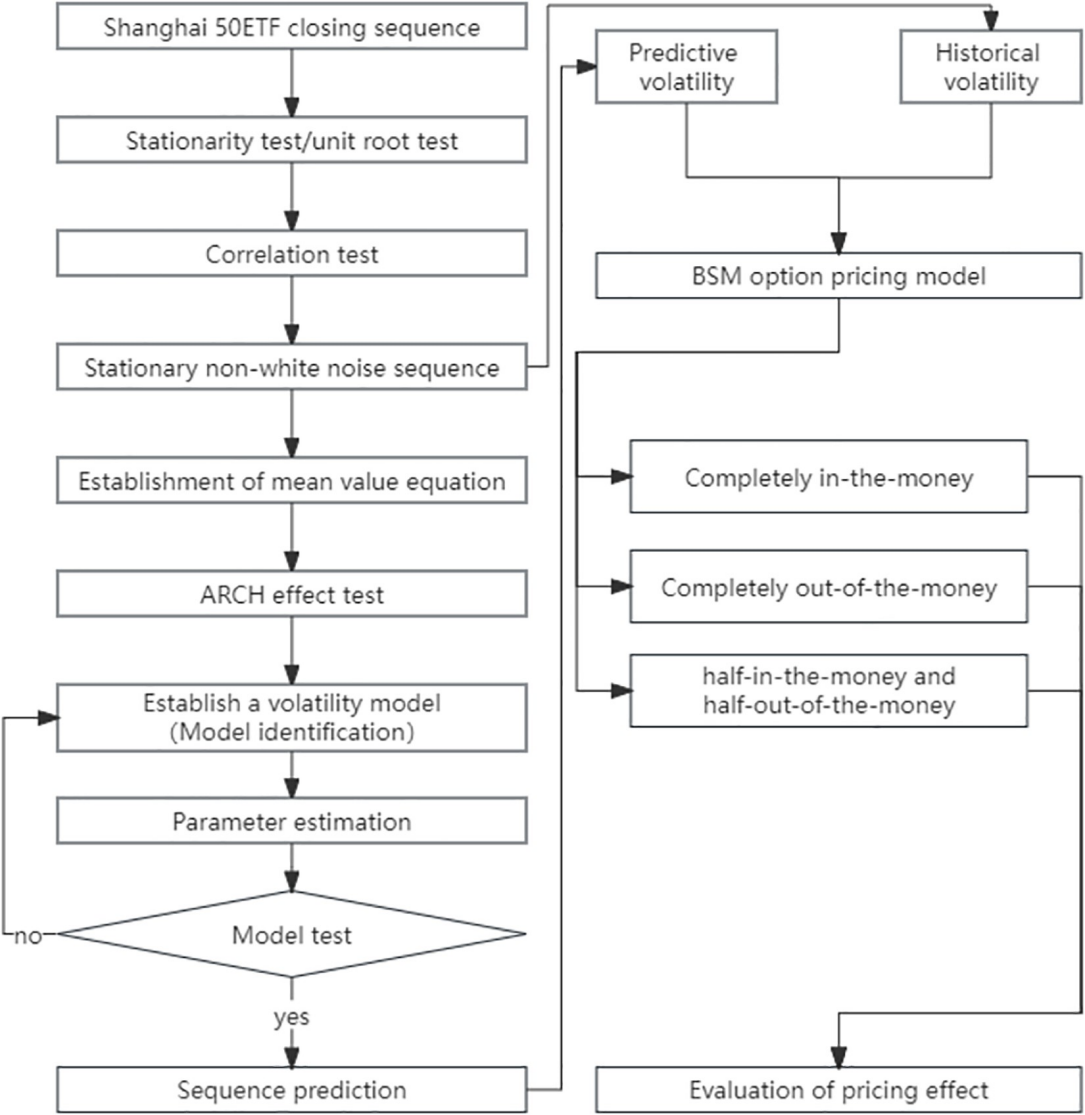
Analysis framework.

Based on the theoretical knowledge of the above model, the empirical analysis of Shanghai 50ETF option contract pricing is conducted. In the empirical process of data preparation, model preparation, parameter estimation, and model diagnosis, the method and results are described in details, which can ensure the reliability and repeatability of the analysis. Fig 1 shows the general flow of empirical analysis:

#### Descriptive statistics

We selected the SSE 50ETF option contract that expired in April 2022 and the option contracts with strike prices of 2.5,3.0 and 3.5 to test the pricing effect of the pricing model under different value states. The selection of option contracts mainly follows the following three principles:

The first principle is the value status of SSE 50ETF option contracts. We collected all option contracts listed since February 2015, and screened them in reverse chronological order. The reason is that it is of no practical significance to study the pricing of option contracts dated too long ago.

The second principle is the relationship between the exercise price and the underlying price of the option. Options can be divided into real option, virtual option and semi-real and semi-virtual option. In order to test the generalization ability of the model pricing, option contracts in these three states are selected for research.

The last principle is the term of the option contract. The terms should not be overly long or short because overly long or short model prediction steps can lead to inaccurate results and problems.

Therefore, the complete sample interval of the SSE 50ETF closing price data is from 2015-2-9 to 2022-4-27, a total of 1,755 trading days. The interval division of the training set and a test set of volatility modeling prediction is shown in Table 1 and descriptive statistics are shown in Table 2. Therefore, it is only necessary to conduct descriptive analysis and correlation tests for the whole sample during volatility modeling. The GARCH and APARCH models are used to predict the volatility required for option pricing. The closing price data comes from wind and is obtained through the R language.

**Table 1:** Data selection interval.

pricing Target	Volatility training set	Volatility test set and pricing range
Expired in April 2022	2015.02.09–2022.02.23	2022.02.24–2022.04.27

**Table 2:** Logarithmic yield statistics of Shanghai 50ETF.

	Sample size	mean	Standard deviation	skewness	kurtosis	JB	P values
Set of training	1712	0.0002	0.0147	-0.5393	7.4625	4068.7	2.2e-16
Set of tests	43	-0.0031	0.0181	-0.3840	1.0682	3.9974	0.1355

The pricing formula of the BSM model involves parameters such as the spot price of the underlying asset St, the strike price K, time to maturity (T-t), the continuous compounding risk-free interest rate r, the dividend yield q and the annualized variance. The values of these parameters are described as follows:

1. The closing price of the SSE 50ETF corresponding to each trading day is selected as the price of the underlying asset, and the data is obtained from Yahoo Finance through R language;
2. Expiration was in April 2022: strike price K = 2.5, 3.0, 3.5;
3. The quotation of the Shanghai interbank offered rate (SHIBOR) has a high bank credit degree, an excellent quotation mechanism, open and transparent daily data, diverse interest rate variety, and a reasonable term structure. In this sense, SHIBOR as the benchmark interest rate is of a wide range and a strong base. Therefore, one-year SHIBOR is selected as the alternative value of risk-free interest rate r and dividend yield q;
4. There are 252 trading days a year, so the remaining term translated into years is the number of days left in the option contract /252.

**Fig 2:**
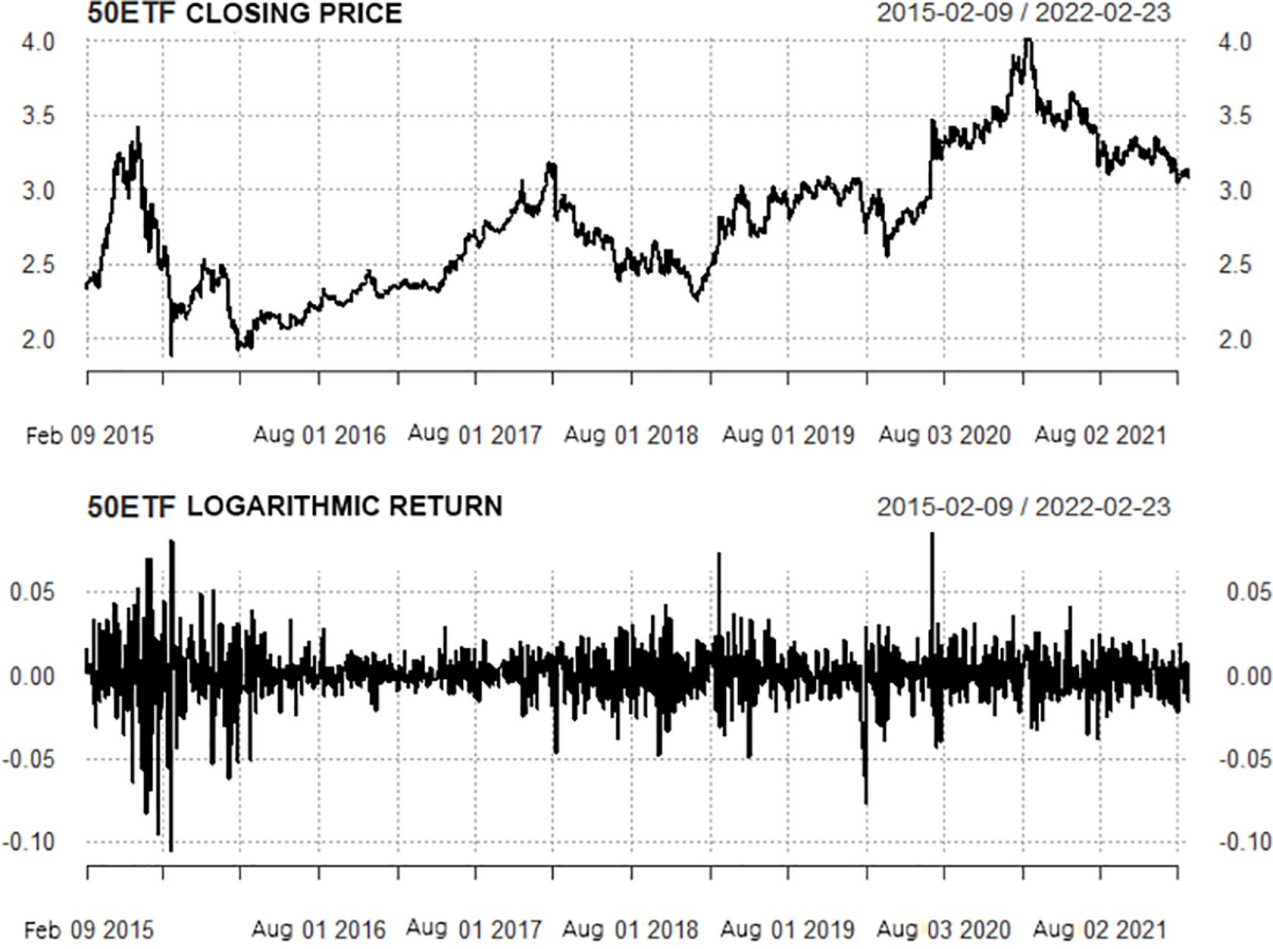
Sequence diagram of closing price and logarithmic yield of the SSE 50ETF.

**Fig 3:**
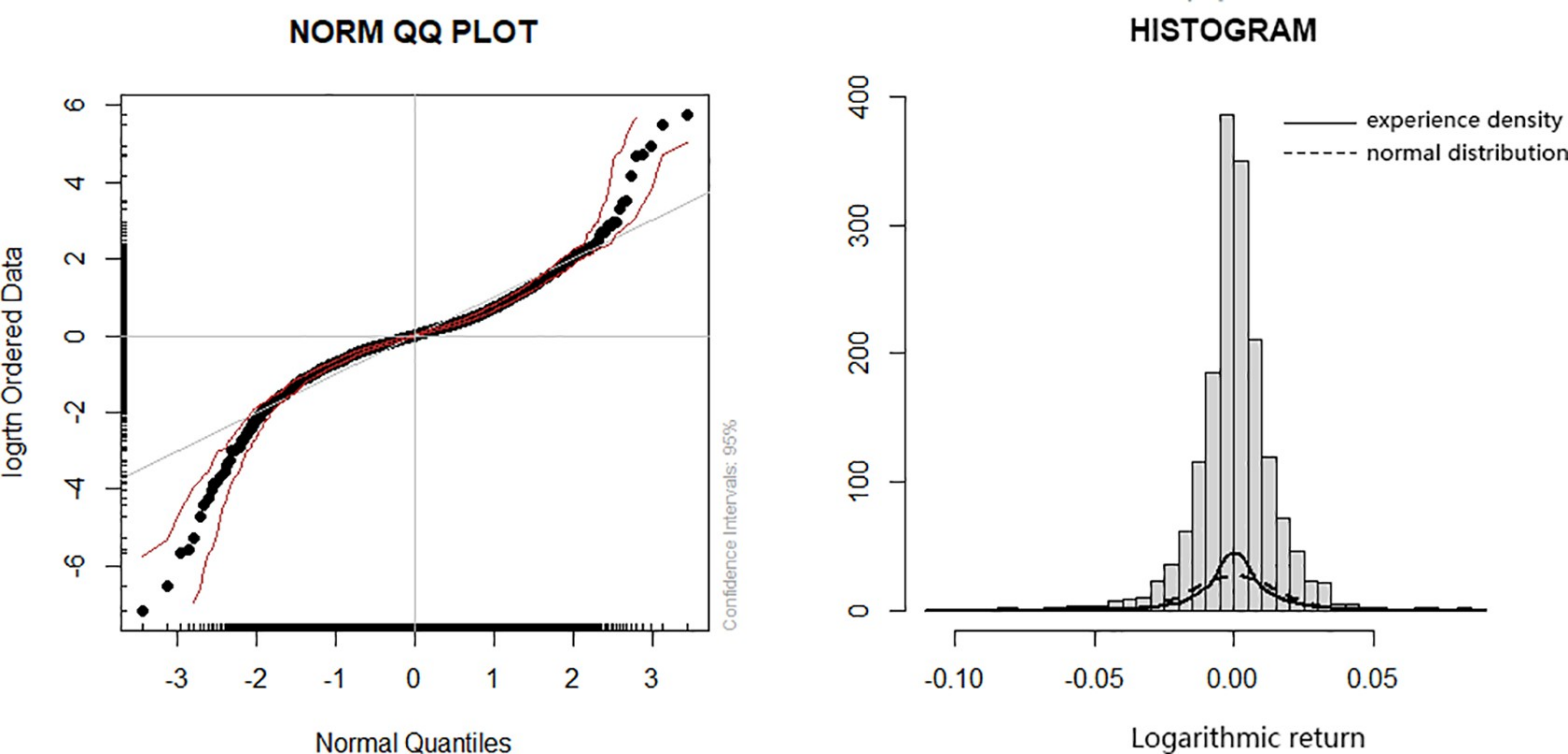
Logarithmic return sequence histogram and Q-Q chart of the SSE 50ETF.

It can be seen from the closing time sequence diagram of the SSE 50ETF in Fig 2 that the sequence has an apparent upward trend, and the sequence is not stable. Since the research object is option price, there is no negative number before log-difference processing. And the log-difference processed sequence can achieve the following three goals: representing the relative change between adjacent time points, objectively reflecting the rise and fall of its return rate, and eliminating the non-stationarity of the sequence. As a result a smooth characteristic of the sequence can be realized, which makes it easier to establish the ARMA-GARCH model. Therefore, we take logarithm and difference of the original time series to obtain the logarithmic return series.Therefore, it is necessary to take logarithmic and differential processing for the closing price sequence to obtain the return sequence of the logarithmic number. The logarithmic return time sequence diagram in Fig 2 clearly shows that the volatility of the return rate is obviously different in different time periods. This indicates that the variance of the return series is time-dependent and immediate; At the same time, there is the phenomenon of volatility clustering. The mean value of the logarithmic return training sample of the SSE 50ETF is 0.0002; the standard deviation is 0.0147; the skewness is equal to -0.5393, less than 0; it has a left-skewed trend; the kurtosis is equal to 7.4625, which is much larger than 3. Combined with the histogram and Q-Q chart in Fig 3, the logarithmic return series of the SSE 50ETF presents the peak and thick tail distribution characteristics. The J-B statistical value of the training sample is 4068.7, and the P value is close to 0, indicating that the null hypothesis that the sequence conforms to normal distribution should be rejected.

#### Relevant inspection

*Stationarity and unit root test*. The statistical rule of a stationary time series does not change with time. If the mean and variance of the return do not change with time or have time invariance, we say the logarithmic return is weakly stationary. According to the time sequence diagram in Fig 2, the return rate series fluctuates around the value of 0, and there is no obvious trend. It is preliminarily judged that this is a stable time series. However, this graphical method is subjective and arbitrary, and we need rigorous statistical tests to confirm it. ADF and KPSS tests were performed on the sequence, and the test results are shown in Table 3. The P value of the ADF test was less than 0.05, rejecting the null hypothesis of the existence of the unit root. The statistics of the KPSS test are all smaller than the critical values of each significance level, indicating that the null hypothesis of the existence of the unit root should be rejected. To sum up, the logarithmic return series of the SSE 50 ETF is a stationary time series. Therefore, applying the GARCH and APARCH models is effective for volatility modeling.

**Table 3:** ADF test and KPSS test results.

ADF test		KPSS test					
statistics	p-value	statistics	significance level	10%	5%	2.5%	1%
-12.354	0.01	0.0383	Critical value	0.347	0.463	0.574	0.739

**Fig 4:**
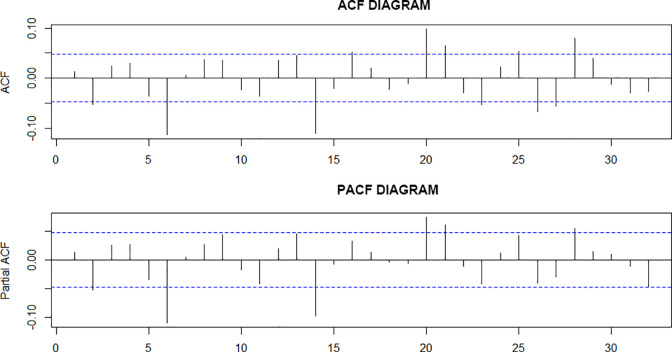
ACF and PACF charts of the logarithmic return series of the Shanghai 50ETF.

*Sequence correlation test*. R language was used to conduct a correlation test on the return rate series, and the results are shown in Fig 4. The dashed line was the interval of the estimated standard deviation of plus or minus twice. The autocorrelation coefficient and partial autocorrelation coefficient of the logarithmic return series displayed in the diagrams indicate the characteristics of trailing and oscillation. Therefore, it can be determined that the logarithmic return series has high-order autocorrelation, and it is necessary to establish the ARMA(p,q) mean equation.

**Fig 5:**
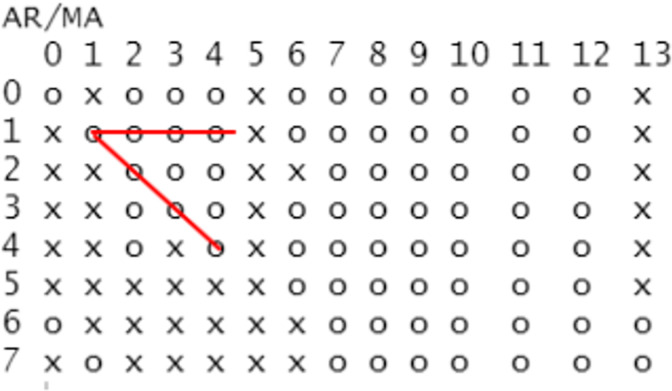
EACF diagram of the logarithmic return series of the SSE 50ETF.


*Establish the mean value equation*. The order determination of the ARMA(p,q) model can be carried out with the help of the EACF diagram. The ARMA(P,Q) model is used to determine the ARMA order by using the criterion that there is a triangular pattern of zeros in the EACF theory. The EACF diagram in Fig 5 shows that ARMA(1,1) as the top angle forms exactly a triangular pattern consisting of zeros, and the constant term of ARMA(1,1) is approximately zero and not significant by a preliminary test, which needs to be eliminated.Therefore, we finally established the mean value model of ARMA(1,1) without constant terms. The significance of parameter estimation is judged according to whether the estimated values of parameters fall between the standard error of two times positive and negative. The results show that all the parameter estimates are significant and that the model-fitting effect is good. Therefore, we can obtain the ARMA(1,1) model:

$$\begin{array}{c} r_{t}=-0.864r_{t-1}+0.892a_{t-1}\\ \left(‐10.523***)\;(12.140***\right) \end{array}$$ (6)

*ARCH effect test*. For testing the ARCH effect, the first method is to carry out the autocorrelation Ljung-Box test on the residual square of the mean equation. The second method is to carry out a LaGrange multiplier (ARCH-LM) test on the residual of the mean value equation. Table 4 shows the ARCH effect test results, which demonstrate that whether the Ljung-Box test or the Arch-LM test, their P values are close to 0 in order 1, 6 and 12 lag. This is to say that the null hypothesis of the absence of the ARCH effect should be rejected. These tests all indicate a powerful ARCH effect on the residual sequence.

**Table 4:** Test results of the ARCH effect.

Lags	Ljung-Box test		ARCH-LM test	
	X-squared	P values	Chi-squared	P values
6	26.023	0.0002205	195.57	< 2.2e-16
12	36.428	0.0002764	212.09	< 2.2e-16
24	94.972	2.134e-10	295.07	< 2.2e-16

The results of the above tests on the peak and thick tail, stationarity, series correlation and ARCH effect of the return series illustrate that it is practical to use the GARCH model and APARCH model to describe the volatility of the logarithmic return of the SSE 50ETF.

*Estimation of volatility model parameters*. As is known above, the logarithmic return sequence has passed the ARCH effect test. According to the PACF diagram of the residual square sequence of the mean model, it is necessary to establish a higher-order ARCH model. In order to make the model simple, we apply the GARCH(1,1) model. At the same time, we need to establish the APARCH δ = 2 (1, 1) model to deal with the volatility of the leverage effect. The mean value equation determined above is ARMA(1,1). Therefore, the ARMA(1,1) -GARCH (1,1) model and the ARMA(1,1) -APARCH (1,1) model will be established for the logarithmic return rate of the SSE 50ETF. After repeated trials, we found that the constant terms of the mean value equation and the auto-regressive heteroscedasticity equation are significantly approximate to 0. Hence, the constant terms need to be removed from the model. The adjusted estimation results of the model parameters are shown in Tables 5 and 6.

**Table 5:** Parameter estimation results of ARMA(1,1)-GARCH(1,1) model.

	Parameter estimation value	Standard error	T statistic	P values
AR(1)	-0.731	0.156	-4.682	3e-06 ***
MA(1)	0.770	0.145	5.295	0e+00 ***
*α*	0.069	0.007	9.345	0e+00 ***
*β*	0.930	0.007	127.037	0e+00 ***

**Table 6:** Parameter estimation results of ARMA(1,1)-APARCH(1,1) model.

	Parameter estimation value	Standard error	T statistic	P values
AR(1)	-0.732	0.146	-5.006	1e-06 ***
MA(1)	0.773	0.136	5.698	0e+00 ***
*α*	0.061	0.007	9.114	0e+00 ***
*γ*	-0.036	0.040	-0.895	0.371
*β*	0.926	0.008	111.756	0e+00 ***
*δ*	2.432	0.289	8.407	0e+00 ***

**Fig 6:**
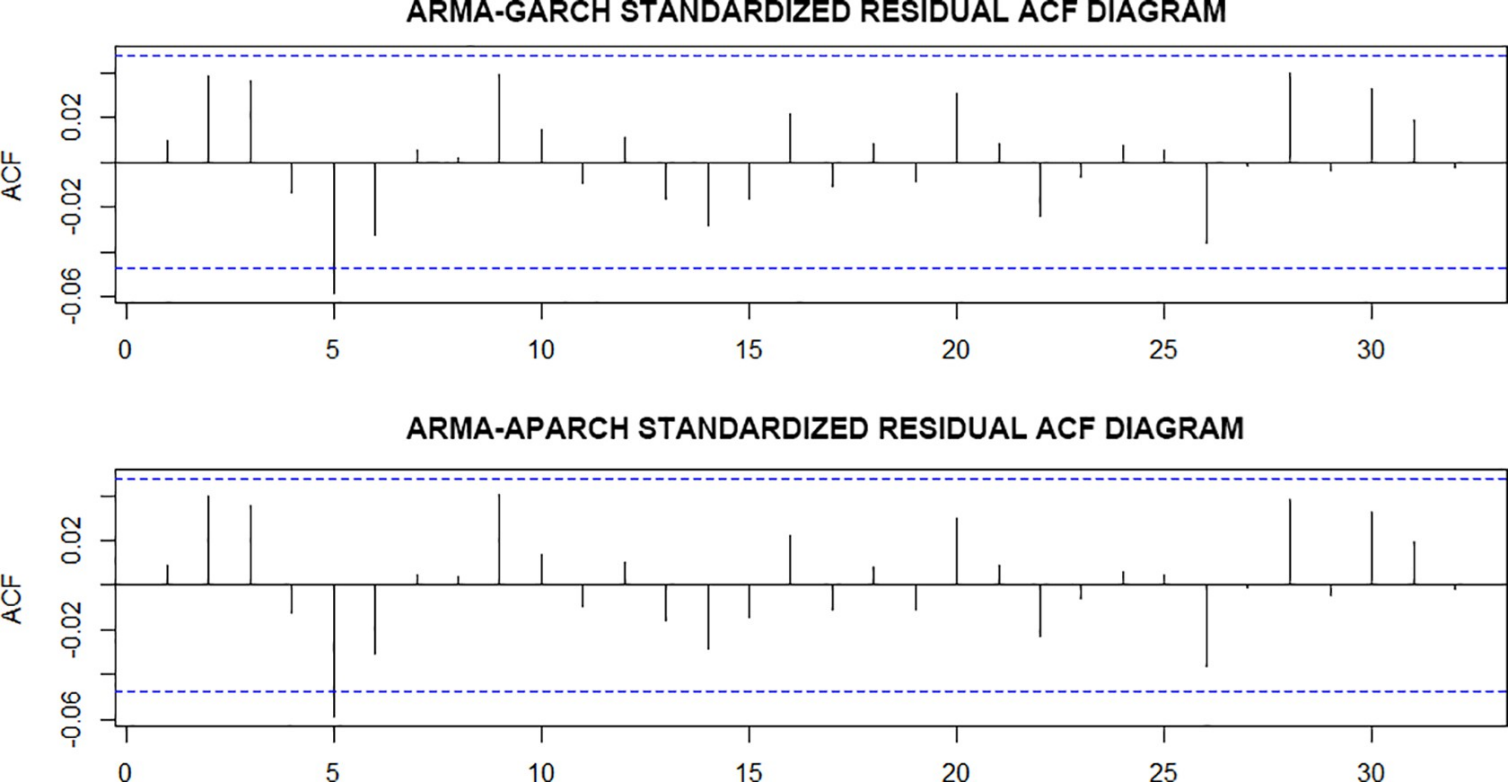
Standardized residual ACF diagram.

According to the parameter estimation results, all the other parameter estimates are significant except for the parameter estimation of the APARCH model, which indicates that the two models have an excellent fitting effect and can describe the conditional heteroscedasticity of volatility. Both models are close to 1, satisfying the model constraints. After the test of the model and the ARCH effect test of the standardized residual of the model, we found the P-values of the Arch-LM test of the model residual are 0.1365 and 0.1308, respectively. Moreover, the ACF diagram of the standardized residual in Fig 6 has implied no auto-correlation with residual sequence. These two test results show that the original hypothesis that the ARCH effect does not exist in residual series is accepted, which means the model test is passed.

Moreover, the information extraction of the volatility model on the logarithmic return rate of the SSE 50ETF is complete and sufficient. It is worth noting that the leverage factor γ APARCH model coefficient estimation was insignificant, showing that the SSE 50 ETF logarithm yield sequence has a weak lever effect because its estimate is not close to 0. Therefore it is maintained to see if weak leverage can improve the prediction accuracy. The APARCH delta estimate is 2.432, significantly different from 2, which suggests a difference between the APARCH model and the GARCH model.

**Fig 7:**
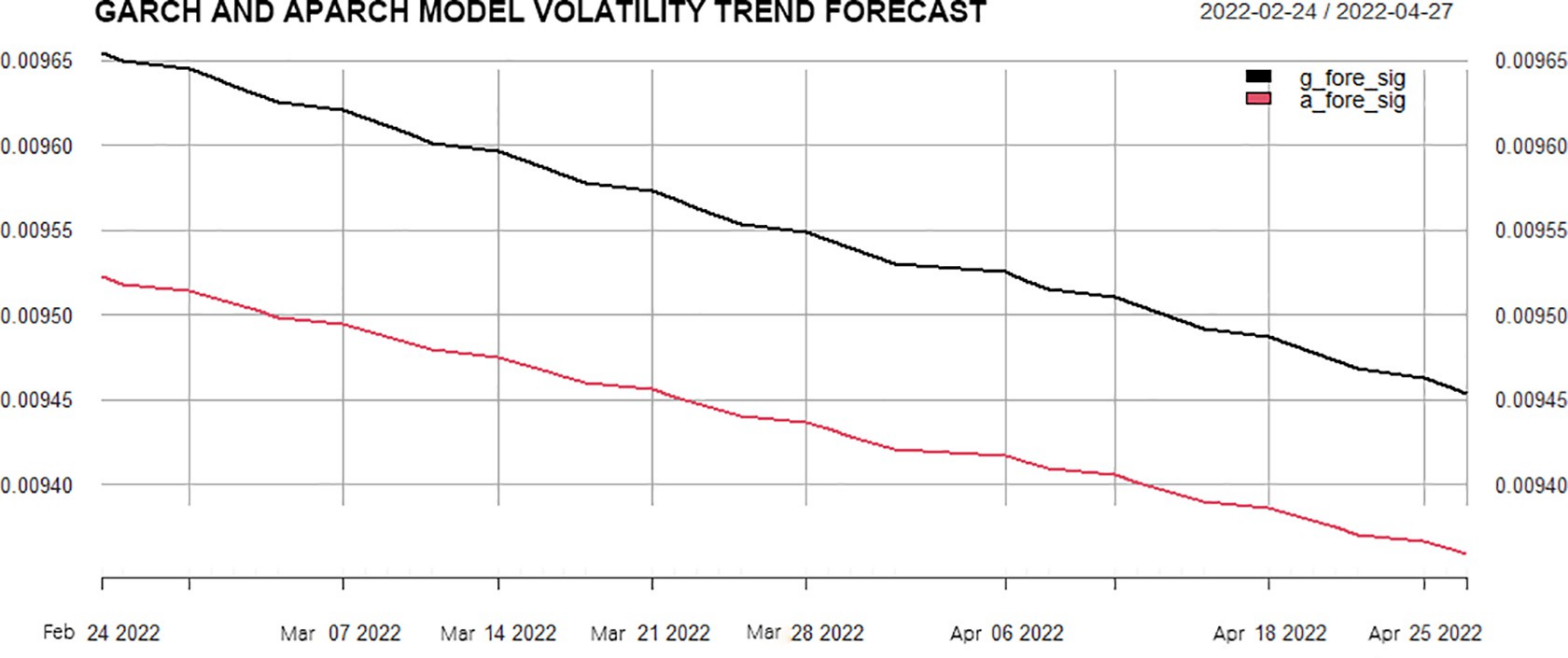
Volatility trend forecast by volatility model.

*Volatility prediction analysis*. In the last section, a volatility model has been established for the logarithm return rate of SSE 50ETF, and the model has passed the test. In order to compare the differences between the two models in predicting volatility, the out-of-sample prediction of the return rate is carried out, and Fig 7 is therefore composed. According to the trend chart, it can be found that the future volatility predicted by the two models has the same trend characteristics. The volatility predicted by the APARCH model with a weak leverage effect is smaller than that of the GARCH model, which reflects the difference between the two models in predicting volatility. It reflects the weak asymmetry of market response to positive and negative price changes, and the reaction to negative price changes is stronger than that of positive price changes. We will investigate their performance in the subsequent option pricing process.

*Analysis of pricing effect*. In this section, we will discuss the pricing of the SSE 50ETF option contract in April 2022 by replacing the volatility predicted above with the constant volatility in the BSM pricing formula for dynamic prediction.

There are two points of understanding about the subscription of put options: first, the option’s value equals the sum of embedded value and the time value; 2. For the call option, when the market price is greater than the strike price, the option contract is in-the-money; otherwise, it is out-of-the-money, and the value state of the put option is the opposite. Given that the minimum closing price of 50ETF between 2022-2-24 and 2022-4-27 is 2.662 and the maximum closing price is 3.105, the call option with a strike price of 2.5 and the put option with a strike price of 3.5 is in a state of completely in-of-the-money. For a put option with a strike price of 2.5 and a call option with a strike price of 3.5 is in a state of completely out-of-the-money., the call option with a strike price of 3.0 is in a state of half in-the-money and half out-of-the-money.

**Fig 8:**
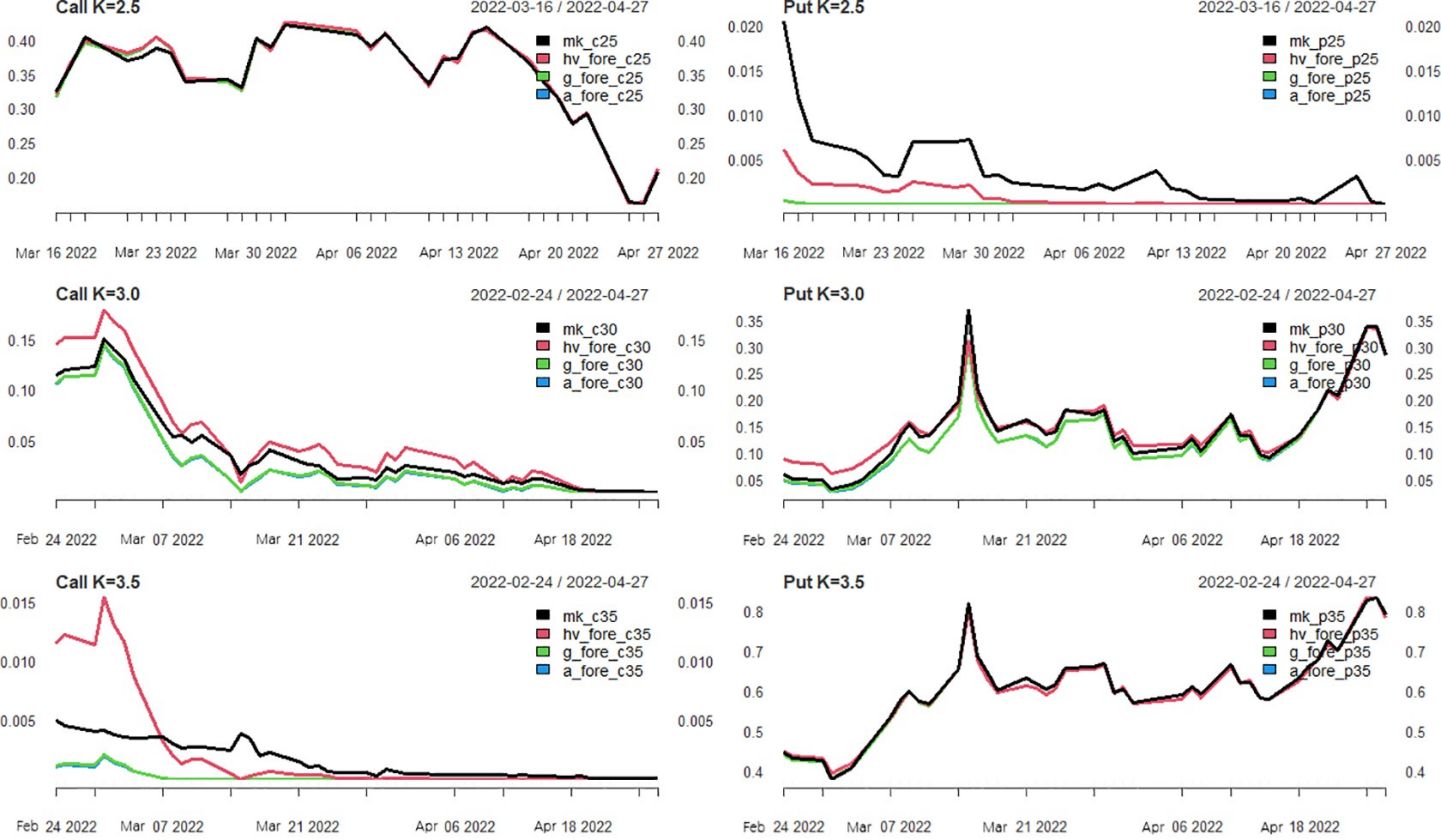
Pricing effect of option contracts expiring in April 2022.

Fig 8 shows the pricing effects of the call put option contracts with three strike prices. Through observation and analysis, we have the following findings: (1) The pricing effect of the completely in-the-money option contract is good, which is very close to the real market price; as the expiration date approaches, the pricing result is closer and closer to the market price. The explanation is that the closing price of SSE 50ETF increases over time in the trading market. Hence, as the expiration date approaches, the embedded value of the in-the-money contract increases while the time value decreases. Then the real market price will be closer and closer to the theoretical price, and the price deduced through the BSM model is the theoretical price. (2) The pricing of the completely out-of-the-money option contract is not ideal. After analysis, it is found that it is because it is out-of-the-money, and its option price only contains the time value, without the embedded value. This is to say that the price of the out-of-the-money option depends on the time value and is greatly affected by time fluctuation. (3) For an option contract with half in-the-money and half out-of-the-money, the pricing effect is not as good as that of completely in-the-money, which means the out-of-the-money state has a negative effect on it. The pricing effect of the in-the-money is better than that of the completely out-of-the-money, that is, the in-the-money loading has a positive effect on it. (4) From the pricing effect of the GARCH model and APARCH model, the APARCH model with a weak leverage effect does not optimize option pricing. (5) For the pricing effects of historical volatility, the GARCH model and the APARCH model to predict volatility, it can be concluded that the pricing effects of the three volatility models are almost the same for the in-the-money option contract, as shown in Fig 8. The pricing effect of the historical volatility model is better than that of the GARCH model and APARCH model, and the pricing effect of the GARCH model is equal to that of the APARCH model. The pricing effect of the historical volatility model and the GARCH model is similar for half-in-the-money and half-out-of-the-money option contracts. Next, we will use some predictive evaluation indicators to make slight measurements of the advantages, disadvantages and differences of the pricing effects of the three volatility models.

The option pricing evaluation indexes in Table 7 are consistent with the observation results in Fig 8: the GARCH and APARCH models are slightly better than historical volatility in completely in-the-money option pricing models. For the completely out-of-the-money option contracts, the volatility predicted by the GARCH model and APARCH model is not as effective as the historical volatility in option pricing. Historical volatility, the GARCH and the APARCH model have sound pricing effects for half-in-the-money and half-out-of-the-money option contracts. In contrast, the historical volatility is significantly lower than the latter. The GARCH model has a slightly better pricing effect than the APARCH model, with a weak leverage effect.

**Table 7:** Options pricing evaluation indexes of different volatility models with different value states.

State of value	Volatility model	MAE	MSE	MAPE	R^2^
Completely in-the-money	Historical volatility rate	0.0060	0.0001	1.2430	99.78%
Completely in-the-money	GARCH	0.0057	0.0001	1.1739	99.80%
Completely in-the-money	APARCH	0.0057	0.0001	1.1747	99.80%
Completely out-of-the-money	Historical volatility rate	0.0023	0.0000	94.4020	-39.24%
Completely out-of-the-money	GARCH	0.0023	0.0000	96.5789	-48.64%
Completely out-of-the-money	APARCH	0.0023	0.0000	96.8894	-49.72%
half-in-the-money and half-out-of-the-money	Historical volatility rate	0.0115	0.0003	31.3219	96.34%
half-in-the-money and half-out-of-the-money	GARCH	0.0113	0.0002	29.7059	96.67%
half-in-the-money and half-out-of-the-money	APARCH	0.0117	0.0002	30.5668	96.51%

## Conclusion

Option pricing has always been a hot topic in financial research, and it focuses on pricing based on the volatility model. This paper selects the first in-market option in China—SSE 50 ETF option as the pricing object. The logarithmic yield of SSE 50ET is used as the volatility modeling object. First, the yield series was used to describe the volatility characteristics, such as peak thick-tail, clustering, sequence correlation, and conditional heteroscedasticity. Then the ARMA-GARCH and ARMA-APARCH models were used to model and predict future volatility. In order to test the effect of the model on predicting volatility, the real market price of options is compared with the theoretical price of model pricing. Finally, the volatility model is evaluated. Based on the above empirical analysis of the volatility model and option pricing research, the following three conclusions can be drawn:

The coefficient of the APARCH model reflects the asymmetry of volatility (leverage effect), that is, whether positive and negative volatility contributes differently to overall volatility. The research results show that the coefficient is negative and small, meaning that negative volatility in the Shanghai 50ETF market has less contribution to the overall volatility, or the market’s reaction to negative volatility is not as strong as that of positive volatility. This indicates that the market is more inclined to positive market trends and less responsive to downside risks. The coefficient also reflects the speed of volatility adjustment, namley the attenuation degree of negative volatility to volatility. This coefficient is a small negative number, indicating that negative volatility has a mild adjustment to volatility and that volatility is relatively stable. This is related to some features in China’s stock market, such as strong market regulation and policy intervention, which may restrain the spread and impact of negative volatility.
(2) The BSM option pricing model combined with the GARCH model has a reasonable pricing effect on the in-the-money option, which is basically consistent with the option market price. The assumptions of the BSM model do not affect its application in practice. However, this pricing scheme does not apply to the pricing of the virtual option because the price of the virtual option is determined by the time value and greatly affected by time fluctuation. As can be seen from the above pricing renderings, this pricing scheme will underestimate the option contract’s value regardless of in-the-money options or out-of-the-money options. Without consideration given to the transaction costs, it is profitable to trade in-the-money options.
(3) In 2021, China’s stock market and the SSE 50ETF suffered a setback due to a continuous impact of the covid-19, which can be verified by the closing price trend of the SSE 50ETF. Regarding the pricing effect of the option contract in April 2022, the BSM option pricing model combined with the GARCH model has an excellent in-the-money option pricing effect, which does not apply to the out-of-the-money option contract. Therefore, the pricing effect of the option is only related to the value state and not affected by economic turbulence. This pricing scheme can stand the test of the market.

We can build a quantitative trading system for in-the-money options based on the above conclusions. The idea is as follows: First, we need to track the closing price of SSE 50ETF in real time to identify in-the-money options, regardless of at-the-money and out-of-money options. Subsequently, we establish the GARCH model on the historical yield data of the SSE 50ETF and predict future volatility. Then the in-the-money option’s theoretical price is calculated using the BSM model. Finally, the theoretical price is compared with the market price, and the trades of option contracts that are overly high or low take place to realize arbitrage. The whole transaction is a real-time, dynamic process that captures every arbitrage opportunity in the market. Similarly, we can use the SSE 50ETF options to hedge with this idea.

### Limitations

This study conducted a complete and rigorous empirical analysis on volatility, leading to a satisfactory proposal of the in-the-money option pricing scheme. However, this study has three main limitations. First, in the Chinese market, where there is an anti-leverage effect, it is still being determined whether the volatility prediction effect of other GARCH family models (EGARCH and TGARCH models) dealing with the leverage effect is similar to that of the APARCH model or not. Second, this paper did not explore the out-of-money option pricing scheme. Therefore, the pricing method of out-of-money options and characteristics and options of the SSE 50ETF is worth further investigation. Last but not least, in terms of option pricing, this paper has broken the limits of the volatility constant of the BSM model, but has not yet corrected other assumptions, such as the normal distribution hypothesis. How to modify the model to improve its accuracy is the direction of the next research.

### Prospect

The research on option pricing based on BSM model combined with ARMA-APARCH model is only an exploratory attempt. There are still many directions for further research. In terms of volatility prediction, first of all, when modeling and forecasting volatility, only one variable, the closing price of Shanghai 50ETF, is used in this paper. The single-variable model prediction is relatively weak. Factors affecting its trend should be taken into account and the combined modeling of multiple related financial variables, such as interest rate, exchange rate and stock price, should be extended. Researchers can explore how to better apply the ARMA-APARCH model to option pricing in a multivariate setting, and study the characteristics of multivariate conditional heteroscedasticity and the interaction of asymmetric effects. Secondly, the data used in this paper is daily data, which belongs to low frequency data. We can explore how to apply the ARMA-APARCH model to high frequency data, such as minute or second data, and study the option pricing problem under high frequency data. In addition, with the advent of big data era, the ARMA-APARCH model can be expanded to high-dimensional data, such as panel data, to adapt to more complex market environments. Finally, there is a sudden jump in volatility in the financial market, which has an important impact on the pricing and risk management of options. Therefore, further research is expected to introduce jump diffusion models, such as stochastic volatility jump model (SVJ) or jump diffusion model (JD), to better describe the volatility jump phenomenon.

## Supporting information

S1 File (ZIP)
